# Is there a stronger willingness to pay for photovoltaic power generation with high education in China?

**DOI:** 10.1371/journal.pone.0296714

**Published:** 2024-04-03

**Authors:** Zhenghua Zhang, Lun Hu

**Affiliations:** School of Economics and Management, Jiangxi Agricultural University, Nanchang, China; University of Maryland College Park, UNITED STATES

## Abstract

Adoption of clean electric energy depends not only on administrative regulations, but also on public support, in particular, the public is willing to pay for environmental improvements. However, the increase of solar photovoltaic power generation willingness to pay (WTP) associated with higher education attainment and the identification of their causality has been missing. Present paper used the enactment of the Compulsory Schooling Law as an instrumental variable to solve the causal relationship between education and willingness to pay for photovoltaic power generation. The results are as follows:Heckman two-stage model and instrumental variable both confirmed that higher education has a positive impact on WTP for solar photovoltaic power generation. For each level of public education in the east, the WTP of photovoltaic power generation will increase by 7.540 CNY, 8.343 CNY and 8.343 CNY respectively, the central public will increase by 9.637 CNY, 10.775 CNY and 11.758 CNY, and the western public will increase by 12.723 CNY, 15.740 CNY and 17.993 CNY respectively. The positive influence of education level is smaller among the people who know the ladder price better, but it is bigger among the people who are male, older than 45 years old, healthier, higher income and stronger awareness of safe electricity use. The total socio-economic value of photovoltaic power generation is significantly different in eastern, central and western region China.

## 1.Introduction

China’s power sector consumes a large amount of fossil fuels every year, and as a result, the power sector has the largest and fastest growing carbon emissions [1]. The large-scale exploitation of fossil energy will cause problems of resource depletion, climate change, environmental pollution and ecological damage [2, 3]. Therefore, on the one hand, China urgently needs to develop clean energy power generation to reduce its dependence on fossil fuels and lower pollutant emissions [4, 5]. In September 2020, the Chinese government formally proposed that "carbon dioxide emissions by 2030; strive to reach the peak, and strive to realize carbon neutrality by 2060 ". At the Climate Ambition Summit, the Chinese government further proposed that "by 2030, the total installed capacity of wind and solar power will reach more than 1.2 billion kw". As one of the renewable energy technologies, photovoltaic power generation has the role of promoting green energy transformation, protecting the ecological environment, and mitigating climate change, and it is an important way for China to realize the goal of carbon peaking and carbon neutrality [6]. On the other hand, there is a need to explore ways to use clean energy from a public perspective, and a fundamental solution is to increase the ability and willingness of the 1.4 billion population to pay more to reduce carbon emissions [7]. Meyer [8] found that education can increase the public’s willingness to pay, and promote the adoption of pro-environmental behaviors by individuals.

After 40 years of reform and opening to the outside world, China has made remarkable progress in public education, with rapid economic development simultaneously promoting significant expansion of the education system, increasing enrollment and literacy rates, and higher education expansion [7] [Higher Education refers to the education implemented on the basis of the completion of higher secondary education, which is to cultivate high-level specialists with a sense of social responsibility, a spirit of innovation and practical ability]. In 1949, China’s literacy rate was only 20%, the net enrollment rate of primary school-age children was 20%, the gross enrollment rate of junior high school level and higher education was 3% and 1.56%, respectively, and the number of students enrolled in higher education nationwide in that year was only 117,000 [9]. On the contrary, in 2020, the net enrollment rate of primary school-age children will reach 100%, while the gross enrollment rates of junior high school, senior high school and higher education will reach 102.5%, 91.2% and 54.4%, respectively [9], and the total number of students enrolled in higher education in 2020 will reach 41.83 million.

Under the pressure of "coal-rich, oil-short and gas-short" in China, the goal of "double-carbon" is forced to be realized to increase the proportion of renewable energy represented by wind and light in primary energy, and vigorously develop renewable energy. According to the relevant research forecast, by the end of 2020, the cumulative installed capacity of renewable energy generation in China will be 934 million kilowatts, an increase of about 17.5% over the same period of last year, accounting for 42.5% of the total installed power; among them, the installed capacity of wind power is 281 million kilowatts, and that of photovoltaic power generation is 253 million kilowatts. At the same time, the national renewable energy power generation reached 2,215.4 billion kWh, accounting for 29.1% of the total power generation; Wind power generation is 466.5 billion kWh, accounting for 6.1% of the total power generation; Photovoltaic power generation is 261.1 billion kWh, accounting for 3.4% of the total power generation. In recent 20 years, China has made great achievements in optimizing energy structure and developing clean energy [4]. However, these energy structure adjustment measures may lead to high energy prices or introduce additional costs for installing new equipment [6], potentially increasing the extra cost of enterprises and consumers [10]. Therefore, the successful promotion and adoption of photovoltaic power generation largely depends on the public’s recognition and cooperation, especially how much the public is willing to pay for the adoption of photovoltaic power generation [11]. Considering that China has a population of 1.4 billion, public support can play a vital role in China’s photovoltaic power generation, because the use of photovoltaic power generation largely depends on the public’s willingness to pay for clean energy.

A large number of literatures have shown that higher education level can promote the adoption of clean energy and increase individual WTP [8]. In fact, the higher WTP reflects the greater motivation of the public to adopt clean energy. If the public WTP is low, the adoption rate of clean energy is low or fails [12]. In order to ensure the success of China’s two-carbon goal, it is necessary not only to investigate how much the public is willing to pay for the adoption of clean energy, but also to study the reasons for increasing individual WTP. The research found that higher economic income [13], younger age [14, 15] and higher education [16] can improve individual WTP. As China is a developing country and a big country in education, this paper attempts to discuss how individual educational achievements can contribute to increasing WTP.

In the past few decades, China’s rapid economic development has promoted the remarkable expansion of the education system, the rising enrollment rate and literacy rate, and the expansion of higher education enrollment [7]. In 1949, the literacy rate in China was only 20% [9]. On the contrary, in 2020, the net enrollment rate of primary school-age children reached 100%, while the gross enrollment rates of junior high school, senior high school and higher education reached 102.5%, 91.2% and 54.4% respectively (National Statistics Bureau, 2021). In 2020, the total number of students in higher education reached 41.83 million. The reason behind the development of this phenomenon lies in the Compulsory Education Law promulgated by the Chinese government in 1986 [7].

In view of this, this paper aims to study the causal relationship between individual educational achievements and WTP photovoltaic power generation. Although a large number of literatures have proved that education has a positive impact on clean energy adopt behavior, this topic has not been fully studied in five aspects. First, in the past, WTP was estimated based on dichotomy model, but it was seldom measured directly by monetary value. Second, the identification of their causality between the two has been ignored, and the endogenous problem between education and photovoltaic power generation WTP has not been solved. Third, the differences between education and photovoltaic power generation WTP in eastern, central and western China have not been analyzed. Fourthly, there is no analysis that education affects WTP of photovoltaic power generation through other potential mechanisms. Fifth, there is no estimation of the socio-economic value of photovoltaic power generation in the whole country, the eastern, central and western regions. Therefore, this paper uses the data of Chinese General Social Survey(CGSS) in 2018 to make a detailed empirical study on the correlation between micro-level education and photovoltaic power generation WTP, focusing on solving the above five problems.

The rest of this paper is structured as follows. The second part introduces the literature review of education and photovoltaic payment, the third part introduces the research methods and data sources, the fourth part reports the model results of heckman’s two-stage regression and instrumental variables, and further analyzes the regional heterogeneity and the econometric verification of the action mechanism. The fifth part reports the estimation of the socio-economic value of photovoltaic power generation across the country and between regions.

## 2.Literature review for solar photovoltaic power generation

Willingness to pay refers to the evaluation of specific services or products by individual consumers, and the evaluation of public goods and environmental products is now widely used [4]. The accurate estimate of WTP of consumers was obtained by CVM [17], and this method used questionnaires to ask respondents the amount they were willing to pay or compensate for non-market goods or services [18]. By the end of 1980s, this method used CO_2_ emission reduction [1]. Nuclear energy [19], coal to gas heating [20], air quality improvement [21], research and development of solar energy [12], improving electric power service [22], rural electrification [23], rural electrification of small power grid [24, 25], off-grid solar energy [26], electricity [27], electric motorcycle [28].

Later, there was little research on WTP photovoltaic power generation in Asian underdeveloped economies [29], only the research on WTP, a renewable energy source, in China [30], India [22], Indonesia [25], Kazakhstan [31], Laos [32], Pakistan [33], However, due to the frequent occurrence of extreme weather, more and more CVM methods are used to study the sustainable development strategy of solar energy [34, 35], photovoltaic power generation is a major form of utilizing solar energy [36], and its potential evaluation, technology optimization and cost calculation are gradually brought into the research field of vision [37]. Meanwhile, the influencing factors of photovoltaic power generation WTP vary from country to country, including social status [38], gender [14], perceived willingness to pay [39], the frequency of payment [40], political party and race [41], awareness of environmental protection [10], renewable type [42–45].

In addition, the identification of their causality between the education reform and public photovoltaic power generation WTP has less, and there are literatures only to solve the causal relationship between education and environmental protection behavior. For example, Meyer [8] finds that education increases the possibility of individuals using environmental protection travel, reduces disposable supplies and energy consumption, and reduces the use of automobiles. The national exogenous variables of primary education are adopted as IV to solve the causal relationship between education and environmental protection travel. Chankrajang and Muttarak [46] also research education will improve the possibility of taking environmental protection actions, such as using cloth bags instead of plastic bags and using energy-saving light bulbs. Higher education contains more knowledge, which is linked with stronger clean energy adoption behavior [47], De Silva and Pownall [48] found that higher education level tends to encourage financial sacrifice for environmental quality. Jin Tianyu [7] thinks that take the promulgation of Compulsory Education Law as an instrumental variable, this paper solves the causal relationship between higher education and willingness to pay for environmental protection. Numata et al. [29] pointed out that the potential mechanism of school education on individual behavior may be that it improves the way individuals deal with new information, has critical thinking skills, or provides the right tools to make more informed behavior decisions.

The above research did not pay attention to the causal relationship and mechanism between higher education and willingness to pay for photovoltaic power generation. In order to solve the deviation of missing variables, this paper adopts compulsory education law as an instrumental variable of education to solve the causal relationship.

### 2.1Basic situation of electric power and energy in China

In 2021, China’s economic and social operation will recover rapidly, and the key contents of energy policy are mainly reflected in improving the capacity of clean energy consumption and support, promoting the development of traditional petrochemical energy to the system reconstruction direction of new energy integration, and guiding documents of market-oriented reform of various energy pricing mechanisms. Table 1 shows the evolution process of China’s power energy policy.

**Table 1:** Overview of legislation, regulations and policies of China’s electric power and energy industry.

content	policy
Legislation and regulations	On December 28, 1995, the Electricity Law was passed.
	On November 1, 1997, the Energy Conservation Law was passed.
	On February 28, 2005, the Renewable Energy Law was passed, which established the mandatory grid-connected system, classified electricity price system, cost sharing system and special fund system. In 2009, the problem of grid-connected consumption of renewable energy power was further solved.
	The State Electricity Regulatory Commission formulated and triggered the "2012 Legislative Work Plan" and proposed the "Electricity Regulatory Law" for the first time.
Policies, strategies and plans	In 2004, the National Development and Reform Commission and others: "Guiding Opinions on Strengthening DSM", No.939 [2004] of NDRC.
	In 2010, the National Development and Reform Commission, etc.: "Power Demand Side Management Measures", Development and Reform Operation [2010] No.2643.
	In 2011, the National Development and Reform Commission and others: "Administrative Measures for Orderly Electricity Utilization", issued by NDRC [2011] No.832.
	In 2012, the Ministry of Finance and the National Development and Reform Commission (NDRC): Interim Measures for the Administration of the Central Government’s Incentive Funds for the Comprehensive Pilot Work of DSM Cities, Cai Jian [2012] No.367.
	In 2015, the National Development and Reform Commission, etc.: Notice on Improving the Electric Power Emergency Mechanism and Doing a Good Job in the Comprehensive Pilot Work of Power Demand Side Management Cities, Development and Reform Operation [2015] No.703.
	2016 National Energy Production and Consumption Revolutionary Strategy (2016–2030), Development and Reform Foundation [2016] No.2795.
	2017 National Development and Reform Commission: Notice on Deepening Supply-side Structural Reform and Doing a Good Job in Power Demand Side Management under the New Situation, Development and Reform Operation Regulation [2017] No.1690.
	National Development and Reform Commission and Energy Administration: Guiding Opinions on Improving the Regulation Capacity of Power System, No.364 [2018] of Development and Reform Energy
	2019 Ministry of Industry and Information Technology: Guide to DSM in Industrial Field, Ministry of Industry and Information Technology Operation [2019] No.145.
	2021 National Development and Reform Commission: Notice on Further Perfecting Time-of-use Electricity Price Mechanism (NDRC Price [2021] No.1093)
	In 2021, National Energy Administration: Notice on Issues Related to the Development and Construction of Wind Power and Photovoltaic Power Generation in 2021 (Guo Neng Fa Xin Neng [2021] No.25)
	2021 National Development and Reform Commission, National Energy Administration: Guiding Opinions on Accelerating the Construction of a Unified National Electricity Market System (NDRC [2022] No.118)
Donor organization	The energy foundation
	Green electric power fund

The above policies can promote the green and low-carbon development of power renewable energy. If the public actively responds to these policies, it can improve the integration of photovoltaic power generation energy in China. But most road maps and policies on renewable energy are implemented without proper understanding of people’s willingness to pay for renewable power and how much they are willing to pay. If we don’t pay attention to this point, China’s photovoltaic power generation will hinder the promotion of clean energy policy because of its high electricity price. Therefore, this paper tries to bridge this gap by determining the cost of photovoltaic power generation that the Chinese public is willing to pay and the regional differences in payment from the perspective of higher education.

### 2.2Introduction of clean energy consumption in China

**Fig 1:**
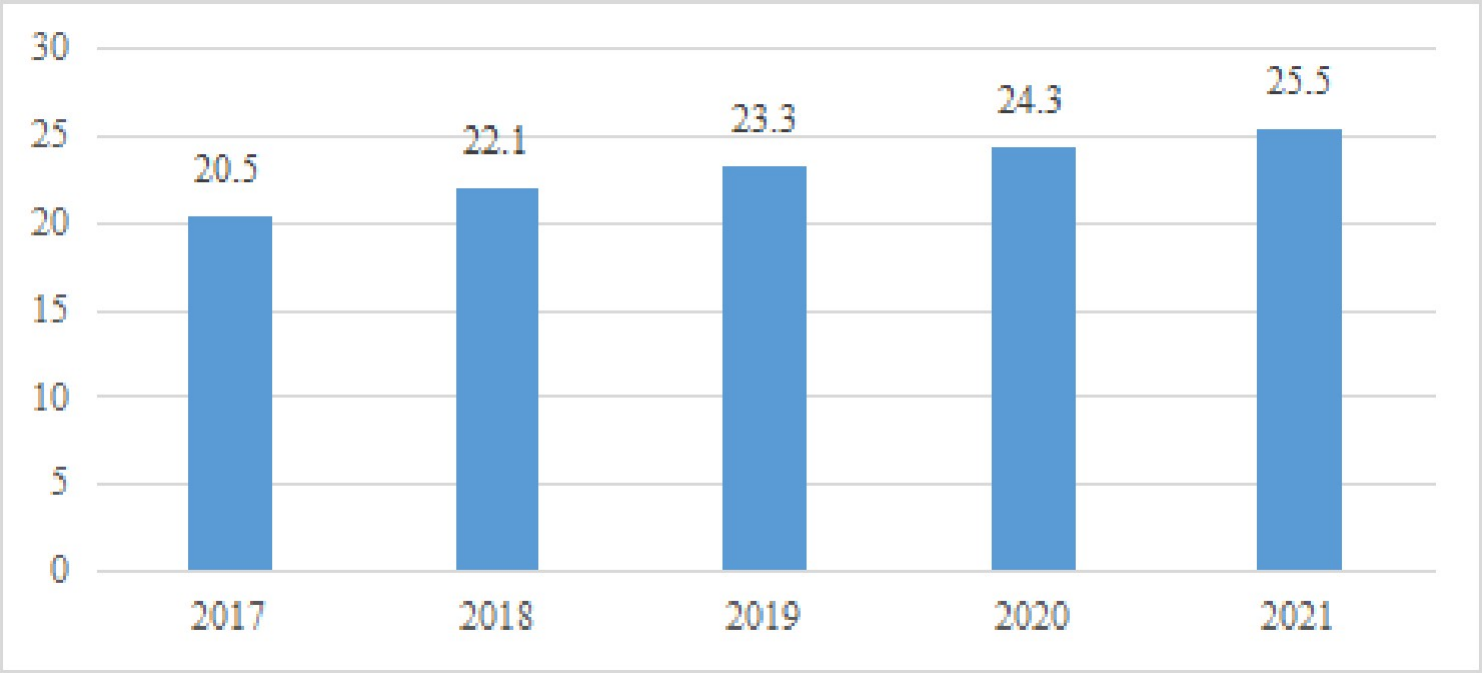
Proportion of clean energy consumption.

**Fig 2:**
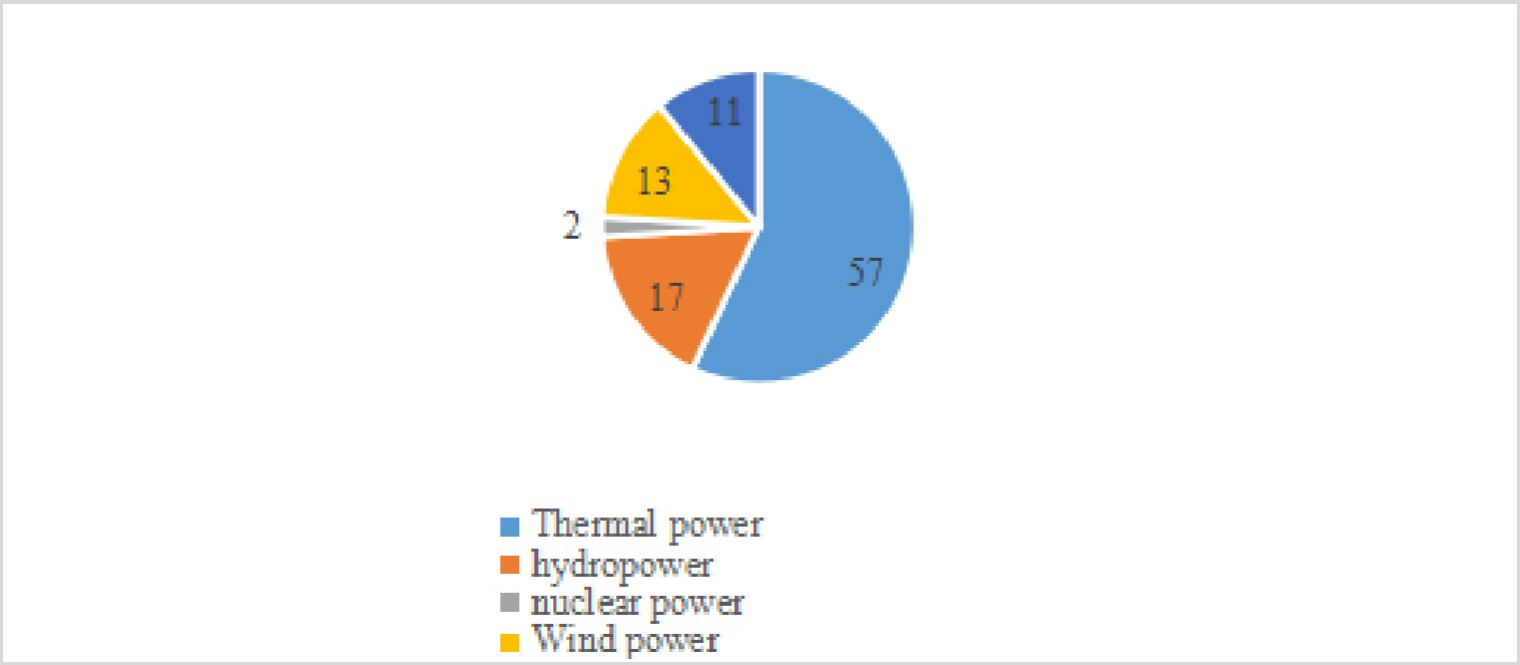
Installed capacity in 2020.

According to the data of China Statistics Bureau in 2022, coal consumption accounts for 56.0% of the total energy consumption, down 0.9 percentage points from the previous year; Fig 1 shows that natural gas, hydropower, nuclear power, wind power, solar power and other clean energy consumption accounted for 25.5% of the total energy consumption, up by 1.2 percentage points. It can be seen that the proportion of disposable energy in energy consumption is gradually decreasing, and the consumption of clean energy is gradually increasing. Fig 2 shows that from the perspective of installed capacity, the installed capacity of wind power PV accounts for about 24%, which exceeds 17% of hydropower. Fig 2 shows the power generation. The photovoltaic power generation of wind power only contributes 9% of the total power generation, which is half of the power generation contributed by hydropower.

**Fig 3:**
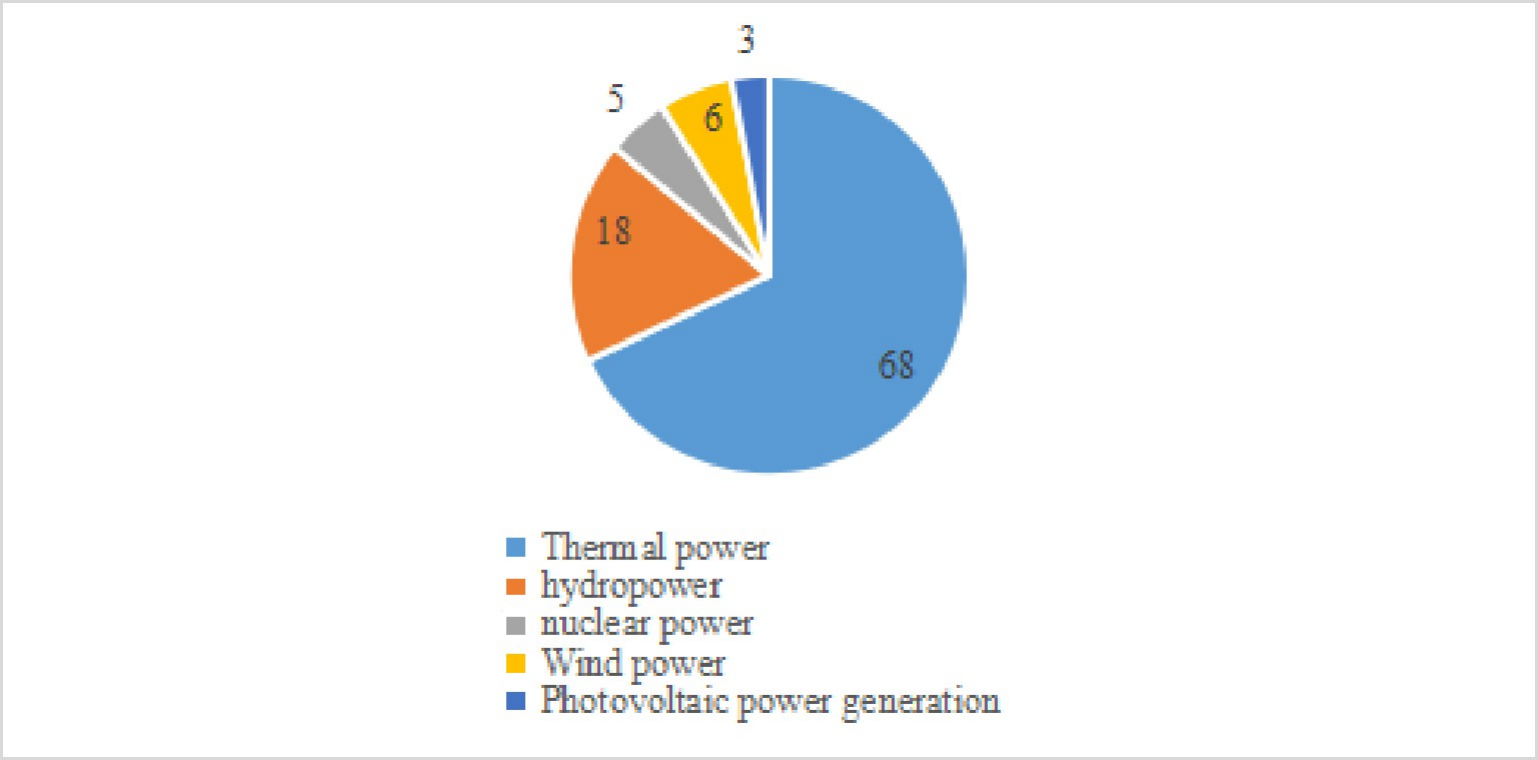
Power generation in 2020.

It can be seen from Fig 1 shows that the public’s demand for clean energy consumption is on the rise, The main reason for the increase in new energy consumption is the government’s support for clean energy, which includes the following measures: firstly, encouraging private investment. Including the establishment of special funds, tax exemptions and other measures to encourage private investment in clean energy projects and reduce the cost of clean energy. Secondly, establish preferential policies for clean energy. Provide preferential policies such as tax reduction and increase in grid electricity prices for clean energy power generation enterprises, encourage traditional energy enterprises to make changes, and promote the growth of new energy enterprises. Finally, implement binding goals. The government has set a series of binding targets for carbon dioxide emissions, promoting renewable energy, limiting the quantity of high energy and high polluting products produced and sold afterwards. The above government’s support measures for clean energy have promoted a surge in demand for new energy consumption, playing a very positive role. Fig 2 shows that the installed capacity of PV is only 11%, and Fig 3 shows that the share of wind power and PV in total power generation is only 9%, from which it can be introduced that the growing public demand for clean energy consumption and the low installed capacity and insufficient power generation of clean energy contradict each other prominently, which drove the government to vigorously promote PV power generation to meet the consumers’ demand for clean energy consumption. Therefore, it is necessary to know the public’s willingness to pay for wind power and photovoltaic power generation, and help investors and the public to reflect the low-cost installed capacity and high-efficiency power generation.

### 2.3Data of China’s electricity restriction practice in January-May 2021

Table 2 shows the structure of China’s power generation in the first five months of 2022. From the power supply side, the power generation of hydropower and photovoltaics in the first five months of 2022 increased rapidly (including 17.5% for hydropower and 24.4% for photovoltaics), but the thermal power also decreased by 3.5%. It shows that at present, China’s clean energy, especially hydropower and photovoltaic power generation, is growing rapidly.

**Table 2:** Value of China’s power generation in the first five months of 2022.

	Electricity generation (TWh) is 100 million kilowatts	Year-on-year growth rate (%)
**Gross generation of electricity**	32484	0.5
**Thermal power generation**	22712	-3.5
**Water power**	4346	17.5
**Wind power generation**	3256	9.5
**Nuclear energy power generation**	1663	4.5
**Photovoltaic power generation**	3.2	24.4

From the perspective of clean development, in the first five months of 2022, hydropower and photovoltaic power generation grew rapidly, wind power was basically stable, and nuclear power generation was also growing in an orderly manner, and the trend of clean energy development remained unchanged on the whole. However, with the rapid growth of power demand, although the growth of clean energy (including wind power and photovoltaic power generation accounting for 9%) can’t meet the increase of power demand, coal-fired power generation declines and the cleanliness of the overall power structure increases. In order to better summarize and think about the phenomenon of power rationing in 2021 and fully and profoundly understand the law of low-carbon transformation of power system from the demand dimension, it is necessary to measure the willingness to pay for photovoltaic power generation (especially wind power and photovoltaic power generation) from the public perspective.

## 3. Materials and methods

### 3.1 Research methods

#### 3.1.1 Heckman two-stage regression model

In order to reduce the potential selection bias, heckman solved the self-bias of sample selection in two stages [49]. Heckman’s two-stage model involves two equations, namely the selection equation and the result equation. The selection equation uses Probit model to estimate the probability of whether the public is willing to pay for solar photovoltaic power generation. The independent variable function is as follows:

$$\text{Prob}\left(\text{D}=1|\text{Z}\right)=\varphi \left(\text{Z}\varepsilon \right)$$ (1)

D virtual variables represent WTP (D = 1 indicates whether the public is willing to pay for photovoltaic power generation, D = 0 indicates unwilling to pay, Z indicates explanatory variables, ε are vectors of unobserved factors). Then, the result of model probability estimation can be used to predict the possibility of each public answering WTP photovoltaic power generation questions. The residual of the selected equation is used to construct the selected inverse Mills ratio (λ). When λ is significant, prove that the sample is self-selected. In this case, it is necessary to add an additional λ independent variable to the two-stage OLS estimation to correct the sample selection bias. The equation is as follows:

$${\text{WTP}}_{\text{i}}=\beta_{0}+\beta_{1}{\text{E}}_{\text{i}}+\beta_{2}{\text{X}}_{\text{i}}+\beta_{3}\lambda +\mu$$ (2)

WTP_i_ is the dependent variable of the model, which i means WTP_i_ the response of the first public to photovoltaic power generation. E_i_ is the education that core independent variable in this paper, *β*_1_ is the coefficient of educational status, X_i_ includes the control variables of public characteristics. The demographic characteristics included in this study include gender, age, health level, marital status, family size and monthly electricity consumption. Drawing on the previous research results, we found the cognition of photovoltaic power generation [50], electricity performance and government regulation of energy [51] has a positive impact on public WTP.

#### 3.1.2 IV instrumental variable method

There may be an endogenous causal relationship between the education level and the public’s willingness to pay for photovoltaic power generation due to unobservable missing variables (such as ability). Therefore, this paper adopts the exogenous shock variable of China Compulsory Education Act as the instrumental variable of education level. The reason for choosing nine-year compulsory education as an instrumental variable is twofold: first, because the implementation of the nine-year compulsory education policy is mandatory in China, which stipulates that all school-age children (6 years old and above) must receive nine years of compulsory education, during which tuition and miscellaneous fees are exempted, so that children who would otherwise drop out of school continue to stay in school, thus increasing their years of education, nine-year compulsory education is correlated with the endogenous explanatory The nine-year compulsory education is correlated with the endogenous explanatory variables. Second, the implementation of the nine-year compulsory education policy is not affected by an individual’s time of birth, personal ability, or family background, so the implementation of this policy is not related to willingness to pay for clean energy and satisfies the exogeneity assumption. In July, 1986, China promulgated the Compulsory Education Law and started to implement nine-year compulsory free education in Beijing, Hebei and other provinces and cities. However, due to the spatial heterogeneity and the differences in economic development levels of various provinces and cities, the latest provinces, such as Tibet, have implemented it since 1994. Therefore, we use the exposure degree affected by compulsory education as a instrumental variable of public education level. See Zhang Xiaomin [52] for the specific algorithm, so I won’t repeat it here.

Instrumental variables are as follows: First, use OLS model to estimate the impact of China’s Compulsory Education Act on the education of respondents:

$${\text{E}}_{\text{i}}=\gamma_{0}+\gamma_{1}{\text{ex}}_{\text{i}}+\gamma_{2}{\text{X}}_{\text{i}}+\tau +\omega$$ (3)

ex_i_ indicates that the exposure degree of the impact of compulsory education ranges from 0 to 1. If the individual is over 15 years old in the year when the compulsory education begins to be implemented, the value is 0, which means that the individual is not affected by the compulsory education act. If the individual is not over 6 years old in the year when the compulsory education law begins to be implemented, the value is 1, which means that the individual is completely affected by the compulsory education act, When the compulsory education law was implemented, the compulsory education exposure index value of individuals aged between 6–15 years old was between 0–1, indicating that they were partially affected by the law.

The formula of the two-stage least square method (2SLS) is:

$${\text{WTP}}_{\text{i}}=\delta_{0}+\delta_{1}\hat{{\text{E}}_{\text{i}}}+\delta_{2}{\text{X}}_{\text{i}}+\tau +\mu$$ (4)

Among them, $\hat{{\text{E}}_{\text{i}}}$ it is the fitting value of the above estimated educational level of interviewees, and the coefficient δ_1_ indicates the average influence of interviewees’ educational level on WTP affected by compulsory education law, and determines the causal relationship between interviewees’ educational level and WTP for public photovoltaic power generation.

### 3.2 Data sources

The data of this paper comes from the Chinese General Social Survey (CGSS). CGSS data is the earliest national, comprehensive and continuous academic investigation project in 2003. It systematically and comprehensively collects data of society, community, family and individual, and summarizes the trend of social change. Multi-stage, stratified and probability-to-scale sampling methods are adopted. In the first stage, districts (metropolitan areas and suburbs of prefecture-level cities, provincial capital cities and municipalities directly under the Central Government) and counties (including county-level cities) are the primary sampling units. In the second stage, streets and towns are used as secondary sampling units. In the third stage, 25 households were sampled in each selected neighborhood or village committee. In the fourth stage, interviews are conducted with family households and one person in each household as the final unit. According to the data of administrative divisions, the whole country (including 22 provinces, 4 autonomous regions and 4 municipalities directly under the Central Government; There are 2,801 districts and counties (excluding Tibet Autonomous Region, Hong Kong, Macao and Taiwan). These districts and counties, as PSU (Primary Sampling Unit), constitute the overall survey, and are specifically divided into five sampling frames: municipal districts of three municipalities directly under the central government (Beijing, Tianjin and Shanghai), municipal districts of provincial capitals, eastern districts and counties, central districts and counties, and western districts and counties.

CGSS published seven groups of annual survey data from 2010 to 2022, including surveys completed in 2010, 2011, 2012, 2013, 2015, 2017 and 2018. This study uses the survey data completed by CGSS data in 2018. In 2018, CGSS completed a total of 12,787 valid samples. The questionnaire survey includes three parts: Part A core module, Part B social network module and Part E energy module.

#### 3.2.1 Variable selection

(1)Willingness to pay for photovoltaic power generation. In CGSS data in 2018, three questions about willingness to pay for photovoltaic power generation are designed respectively. The first question is "How much more are you willing to pay for electricity every month to increase the number of wind power and photovoltaic power generation per 100 kWh of your household electricity to 10 kWh?", expressed by WTP1, among which 963 households answered 0 CNY, accounting for 40.7%. The second question is "How much more are you willing to pay for electricity every 100 kWh to 15 kWh?" according to WTP2, 959 households answered 0 CNY, accounting for 40.6%. The third question is "How much more are you willing to pay for electricity per 100 kWh of your household electricity to 20 kWh?" According to WTP3, 949 households answered 0 CNY, accounting for 40.1%.

The willingness to pay for photovoltaic power generation in eastern China is higher than that in the whole country, central China and western China. The willingness to pay for photovoltaic power generation in eastern China is increased to 20 degrees, and the public has the highest willingness to pay, with an average of 17.34 CNY. Guangdong province in eastern China has the highest willingness to pay, which is 28.44 CNY, 36.06 CNY and 42.04 CNY, respectively. In addition, the WTP of photovoltaic power generation in China is higher than that in central and western China, and the WTP of photovoltaic power generation in western China is the lowest, among which Guangxi province has the lowest willingness to pay, respectively Shanxi province, the central region, has the lowest willingness to pay, which is 7.292 CNY, 6.815 CNY and 7.369 CNY respectively, while Jiangxi province has the highest willingness to pay, which is 16.537 CNY, 17.618 CNY and 18.967 CNY respectively.

(2)Core explanatory variable: the highest education level of the interviewee, see Table 2 for details.

**Fig 4:**
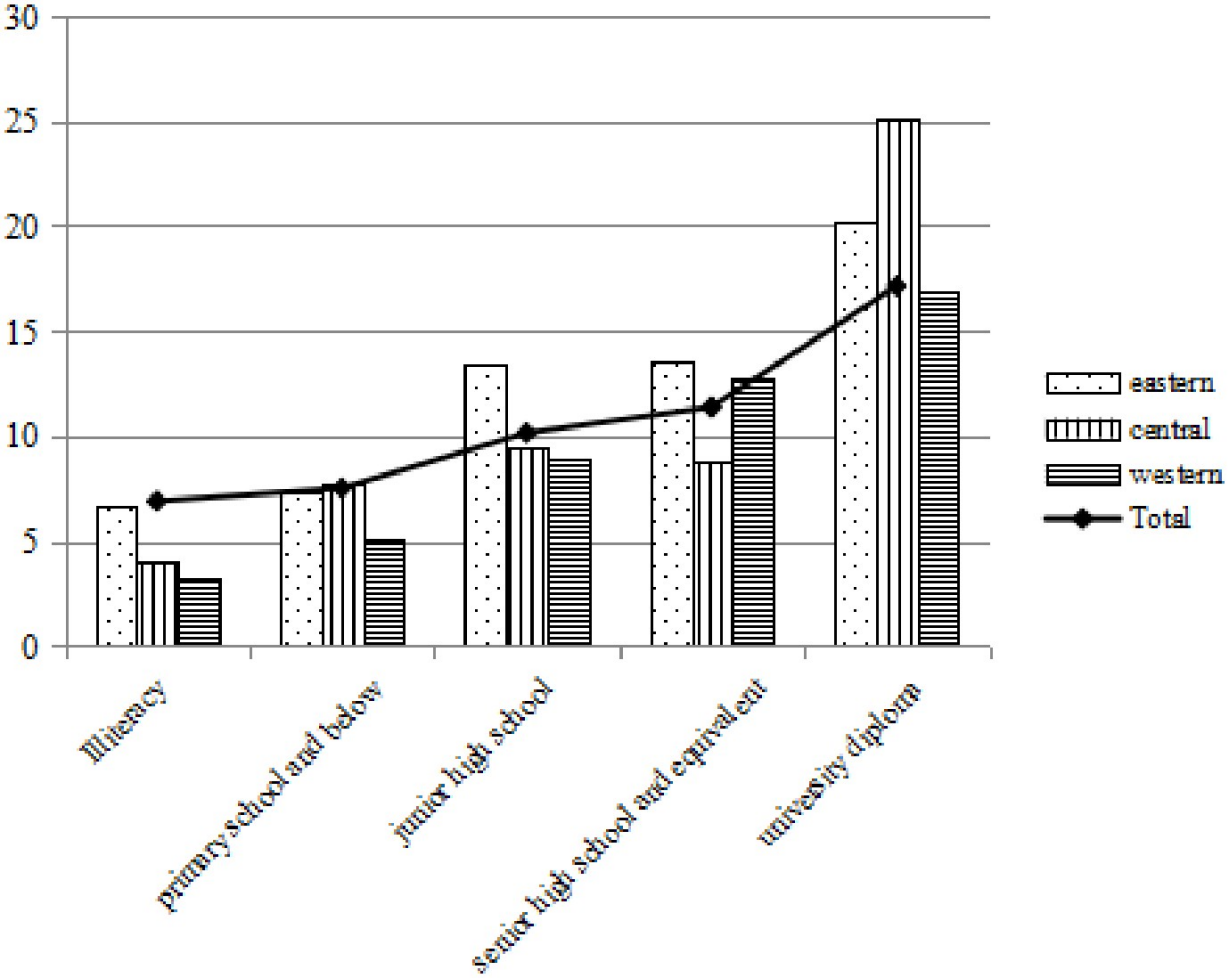
Willingness to pay 10 degrees with photovoltaic power generation at different educational levels.

**Fig 5:**
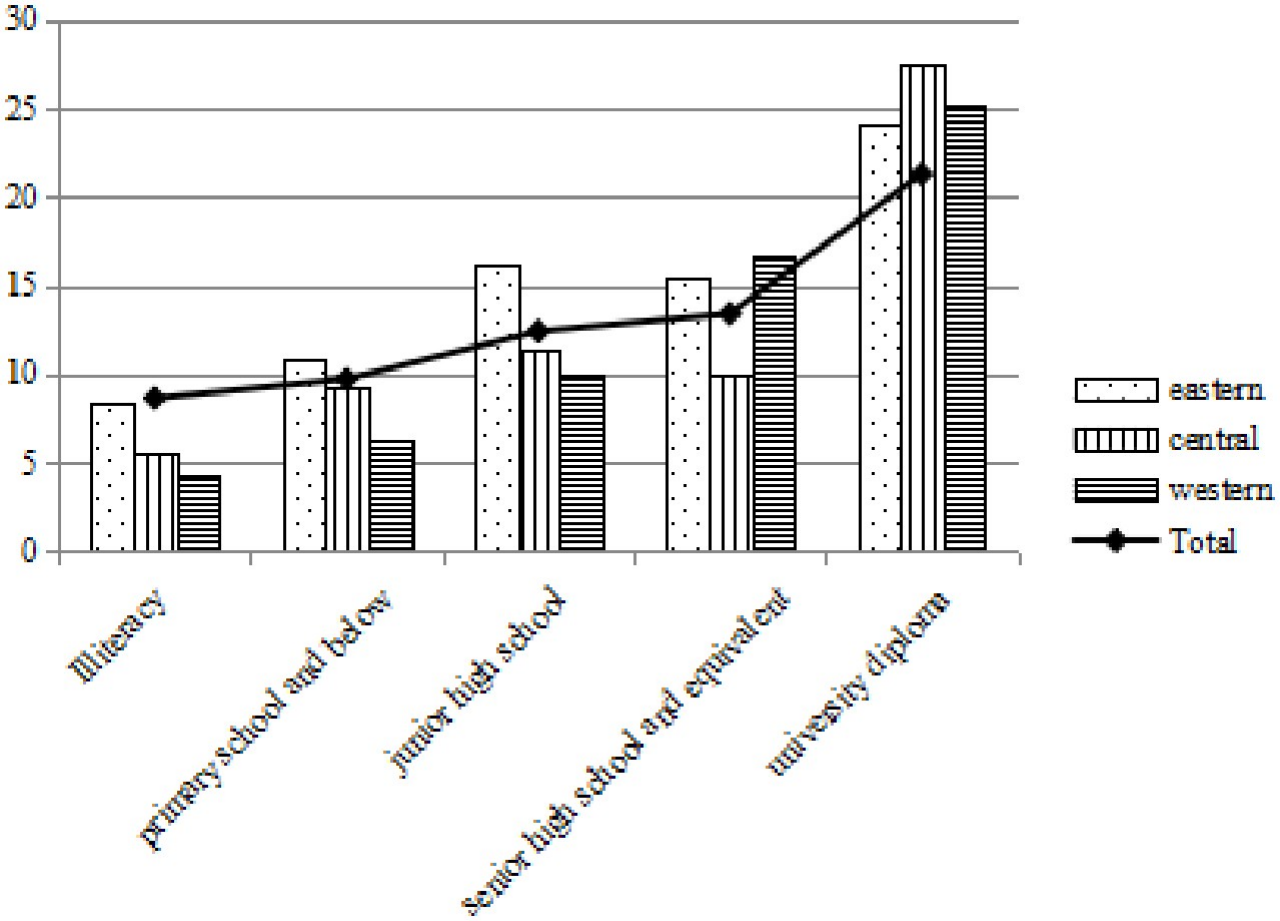
Willingness to pay 15 degrees with photovoltaic power generation at different educational levels.

**Fig 6:**
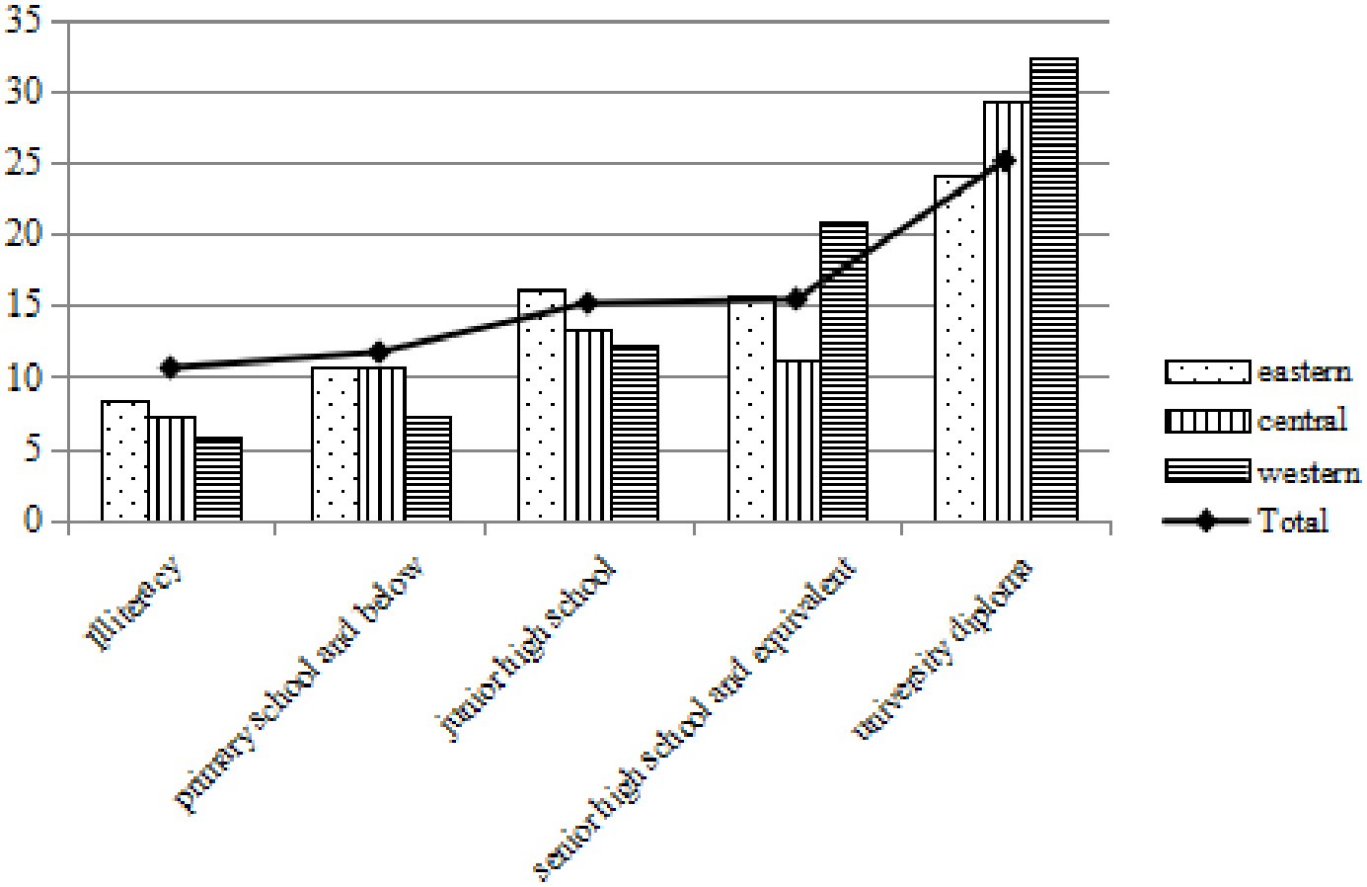
Willingness to pay 20 degrees with photovoltaic power generation at different educational levels.

Figs 4–6 show that under different electricity consumption scenarios, the average willingness to pay for photovoltaic power generation increases with the improvement of education level. No matter in the eastern, central and western regions, higher education has significantly increased the average willingness to pay for photovoltaic power generation. WTP1, WTP2 and WTP3 of photovoltaic power generation in primary schools below are 7.529 CNY, 9.710 CNY and 11.727 CNY respectively, while the average WTP1, WTP2 and WTP3 of undergraduate degree are 17.146 CNY, 21.267 CNY and 25.109 CNY respectively, which are more than twice as high as those of primary school education below.

(3)Other control variables: personal characteristic variables include gender, age, health, marital status, family size, and monthly household electricity consumption/CNY; electricity price awareness includes ladder electricity price awareness, peak-valley electricity price awareness, and photovoltaic power station subsidy awareness; electricity consumption performance includes price rationality, use safety, supply stability, tax increase on energy products to limit consumption, and mandatory policies to limit energy consumption. Descriptive statistics of variables in this paper are shown in Table 3.

**Table 3:** Descriptive statistics of variables.

	Variable	mean	Std.Dev.	Min	max
**WTP1**	How much more are you willing to pay for the monthly electricity bill by increasing the amount of wind power and photovoltaic power generation per 100 kWh of your household electricity to 10 kWh?	10.626	24.547	0	300
**WTP2**	How much more are you willing to pay for the monthly per 100 kWh of your household electricity to 15 kWh?	13.148	28.204	0	400
**WTP3**	How much more are you willing to pay for the monthly per 100 kWh of your household electricity to 20 kWh?	15.764	33.372	0	500
**educational level**	Illiteracy = 1, primary school and below = 2, junior high school = 3, senior high school and equivalent = 4, university diploma = 5, bachelor degree and above = 6.	2.992	1.341	1	6
**gender**	Male = 1, female = 0	0.467	0.499	0	1
**age**	According to the actual survey age/year	55.066	16.179	22	122
**health**	1 = very unhealthy, 2 = relatively unhealthy, 3 = average, 4 = relatively healthy and 5 = very healthy.	3.563	1.067	1	5
**marriage**	Married = 1, unmarried = 0	0.782	0.412	0	1
**Family size**	How many people live in the same family?	2.799	1.381	1	11
**electricity consumption**	Monthly household electricity consumption/CNY	165.54	234.71	0	6000
**ladder price**	You understand ladder price. Not at all = 1, little = 2, average = 3, relatively = 4, and complete knowledge = 5.	2.695	1.341	1	5
**peak and valley electricity price**	You understand peak and valley electricity price.Not at all = 1, little = 2, average = 3, relatively = 4, and complete knowledge = 5.	2.131	1.225	1	5
**photovoltaic power subsidies**	You understand photovoltaic power station subsidies.Not at all = 1, little = 2, average = 3, relatively = 4, and complete knowledge = 5.	1.638	0.838	1	5
**Price rationality**	You rat the electricity price performance.Score the electrical performance of home, 1–10 points.	7.730	5.570	1	10
**Use security**	You rate the electricity security.Score the electrical performance of home, 1–10 points.	8.230	2.361	1	10
**Supply stability**	You rate the electricity supply stability.Score the electrical performance of home, 1–10 points.	8.171	2.389	1	10
**Tax on energy consumption**	Your comments on tax increase and consumption restriction of energy products.Totally disagree = 1, disagree = 2, average = 3, comparatively agree = 4, totally agree = 5.	3.359	0.871	1	5
**restrict energy consumption.**	Your comments on policies to restrict energy consumption.ditto	3.575	0.827	1	5

#### 3.2.2 Descriptive statistics

Table 4 shows that the willingness to pay levels in the eastern region are 14.080, 17.231 and 17.341, which are higher than those in the national sample of 10.626, 13.148 and 15.764 respectively. Similarly, the average value of education water in the eastern region is 3.501, which is higher than the total sample level of 2.992, and the lowest value of education level in the western region is 2.649.

**Table 4:** Comparison of sample areas.

	Total sample		Eastern region		Middle region		West region	
	Mean	Std.Dev.	Mean	Std.Dev.	Mean	Std.Dev.	Mean	Std.Dev.
**WTP**_**1**_	10.626	24.547	14.080	29.525	9.908	28.399	7.980	18.935
**WTP**_**2**_	13.148	28.204	17.231	33.279	11.580	28.737	10.202	24.078
**WTP**_**3**_	15.764	33.372	17.341	33.474	13.239	29.269	12.700	30.687
**educational level**	2.992	1.341	3.501	1.311	2.880	1.210	2.649	1.222
**gender**	0.467	0.499	0.473	0.499	0.465	0.499	0.479	0.500
**age**	55.066	16.179	54.664	16.818	55.592	15.440	54.803	16.369
**health**	3.563	1.067	3.644	1.001	3.586	1.094	3.378	1.109
**marriage**	0.782	0.412	0.788	0.408	0.798	0.401	0.744	0.436
**Family size**	2.799	1.381	2.711	1.331	2.779	1.364	2.992	1.477
**electricity consumption**	165.540	234.705	196.224	314.231	154.301	174.508	132.218	144.278
**ladder price**	2.695	1.341	3.092	1.320	2.505	1.309	2.346	1.247
**peak electric price**	2.131	1.225	2.598	1.346	1.872	1.049	1.791	0.990
**photovoltaic power subsidies**	1.638	0.838	1.768	0.890	1.599	0.785	1.436	0.684
**Price rationality**	7.730	5.570	8.046	6.942	7.334	1.812	8.358	9.105
**Use security**	8.230	2.361	8.251	1.497	8.161	1.444	8.362	4.217
**Supply stability**	8.171	2.389	8.276	1.511	8.084	1.447	8.138	4.291
**Tax energy limit**	3.359	0.871	3.367	0.884	3.354	0.847	3.370	0.861
**Restrict energy**	3.575	0.827	3.622	0.822	3.542	0.808	3.553	0.866
**Sample size**	2364		904		947		513	

Source: 2018 Comprehensive Survey of Chinese Society

The whole sample shows that 35.4% have "comparative knowledge" and "complete knowledge" of the ladder price, and the proportion of "comparative knowledge" and "complete knowledge" in the eastern region is 48.6%; The central part accounts for 29.4%; The western region accounts for 23.2%; It can be seen that the proportion of "comparative understanding" and "complete understanding" of ladder price in the eastern region is higher than the national level.

The whole sample shows that 18.7% have "comparative knowledge" and "complete knowledge" of peak-valley electricity prices, and the proportion of "comparative knowledge" and "complete knowledge" in the eastern region is 22.9%; The central part accounts for 11.7%; Accounting for 8.4% in the west; It can be seen that the proportion of "comparative understanding" and "complete understanding" of peak-valley electricity prices in the eastern region is higher than the national level.

The whole sample shows that 88.9% have "no knowledge" or "little knowledge" about photovoltaic power station subsidies, with 85.7% in the east, 89.8% in the middle and 93.2% in the west. It can be seen that the proportion of "no knowledge" or "little knowledge" about photovoltaic power station subsidies in the west is higher than that in the whole country, the east and the middle.

## 4.Model estimation results

### 4.1 Select bias adjustment

Heckman two-stage regression results are shown in Table 5. It is not difficult to find that the inverse Mills ratio at different levels of willingness to pay has passed the significance test, so it is appropriate to use Heckman two-stage estimation.

**Table 5:** Heckman two-stage regression of WTP.

	WTP_1_		WTP_2_		WTP_3_	
Total sample	Payment equation	Selection equation	Payment equation	Selection equation	Payment equation	Selection equation
**educational level**	0.925**(0.466)	-0.021(0.053)	1.131**(0.526)	-0.020(0.053)	1.402**(0.620)	-0.017(0.053)
**gender**	-0.877(1.072)	-0.146(0.117)	-1.081(1.211)	-0.146(0.117)	-1.581(1.426)	-0.146(0.117)
**age**	-0.033(0.059)	0.270***(0.013)	-0.028(0.067)	0.270***(0.013)	-0.021(0.079)	0.270***(0.013)
**health**	0.973*(0.501)	0.023(0.058)	1.012*(0.565)	0.023(0.058)	1.125*(0.665)	0.023(0.058)
**marriage**	0.306(1.355)	-0.237(0.190)	1.011(1.529)	-0.240(0.190)	1.462(1.801)	-0.246(0.190)
**Family size**	0.211(0.412)	-0.018(0.047)	0.332(0.466)	-0.017(0.047)	0.405(0.548)	-0.016(0.047)
**Electric consumption**	0.003*(0.002)	-0.000(0.000)	0.004*(0.002)	-0.000(0.000)	0.006**(0.003)	-0.000(0.000)
**ladder price**	0.138(0.508)	0.011(0.055)	0.207(0.574)	0.011(0.055)	0.089(0.676)	0.009(0.055)
**peak electric price**	1.280**(0.596)	-0.080(0.064)	1.516**(0.673)	-0.079(0.064)	1.855**(0.793)	-0.080(0.064)
**power subsidies**	1.563**(0.730)	0.002(0.076)	2.283***(0.824)	0.002(0.076)	3.031***(0.970)	0.003(0.076)
**Price rationality**	0.210**(0.093)	0.029(0.023)	0.227**(0.105)	0.029(0.023)	0.233*(0.124)	0.029(0.023)
**Use security**	0.877*(0.506)	-0.103*(0.056)	1.137**(0.571)	-0.103*(0.056)	1.544**(0.673)	-0.103(0.056)
**Supply stability**	-1.103**(0.496)	0.026(0.053)	-1.214**(0.560)	0.027(0.053)	-1.462**(0.660)	0.026(0.053)
**Tax energy limit**	1.264*(0.757)	0.110(0.085)	1.619*(0.854)	0.109(0.085)	2.001**(1.006)	0.107(0.085)
**restrict energy**	-1.151(0.805)	-0.131(0.089)	-1.228(0.909)	-0.130(0.089)	-1.347(1.070)	-0.126(0.089)
**constant**	-1.575(6.320)	-12.897***(0.874)	-5.575(7.133)	-12.902***(0.874)	-9.455(8.411)	-12.911***(0.873)
**Mills Lambda**	2.964***(0.018)	——	3.085***(0.018)	——	3.249***(0.018)	——
No.	2363	2363	2363	2363	2363	2363

Note:

***, ** and * are significant at the confidence level of 1%, 5% and 10% respectively; Numbers in brackets are standard errors.

#### 4.1.1 Influence of education on public willingness to pay for photovoltaic power generation

Table 5 displays the results of the second stage regression show that education level has a significant positive impact on WTP1, WTP2 and WTP3 of photovoltaic power generation at 5% level, indicating that the public’s willingness to pay for photovoltaic power generation has increased significantly with the improvement of education level. The reason for this phenomenon is that. First, a higher level of education means an increase in personal income [53, 54], increasing the ability to pay for photovoltaic power generation. Second, higher education experience may improve public awareness of clean energy [35, 55], and pay more attention to resource and environmental problems such as energy shortage and environmental pollution. Therefore, the public has a stronger willingness to adopt photovoltaic power generation energy to achieve the Pareto optimal state.

#### 4.1.2 Influence of personal characteristic variables on public willingness to pay for photovoltaic power generation

Health has a significant positive impact on public photovoltaic power generation WTP1, WTP2 and WTP3 at the level of 10%, indicating that the healthier the public, the stronger their willingness to pay for photovoltaic power generation. On the one hand, the main reason is that the public’s health shows that they are energetic, have abundant energy to collect knowledge of renewable energy such as wind power and photovoltaic power, and actively adopt renewable energy to reduce negative externalities of resources and environment; On the other hand, the healthy public has a good routine, and it is more necessary to adopt clean energy to reduce environmental pollution and prolong life, so they are more willing to pay for photovoltaic power generation [56].

Monthly household power consumption has a significant positive impact on public photovoltaic power generation WTP1, WTP2 and WTP3, indicating that the more the public consumes monthly power, the higher their willingness to pay for public photovoltaic power generation. The possible reason is that household monthly power consumption represents their higher energy demand, and the government gives policy incentives to low-carbon transformation photovoltaic power generation, which reduces household power consumption cost, thus increasing the public’s willingness to pay for photovoltaic power generation [57].

#### 4.1.3 Influence of electricity price awareness on public willingness to pay for photovoltaic power generation

The understanding of peak electricity price has a significant positive impact on public photovoltaic power generation WTP_1_, WTP_2_ and WTP_3_ at 5% level. It shows that the more the public knows about the peak-valley electricity price, the more willing they are to pay for photovoltaic power generation. The possible reason is that the public knows the peak-valley electricity price, indicating that they have mastered the electricity price level and the price difference ratio in different periods. Usually, the electricity consumption can clearly avoid the peak periods, which can not only save the electricity cost but also use electricity scientifically and reasonably, so the willingness to pay for photovoltaic power generation is strong [58].

The awareness of photovoltaic power station subsidies has a significant positive impact on public photovoltaic power generation WTP1, WTP2 and WTP3, indicating that the more the public knows about photovoltaic power station subsidies, the higher their willingness to pay for photovoltaic power generation. The possible reason is that the more the public knows about the investment cost and subsidy degree of photovoltaic power stations, the lower the cost of photovoltaic power generation, so the stronger their willingness to pay for photovoltaic power generation [59].

#### 4.1.4 Influence of electricity performance on public willingness to pay for photovoltaic power generation

Price rationality has a significant positive impact on public photovoltaic power generation WTP_1_, WTP_2_ and WTP_3_, indicating that the public thinks that the more reasonable the price of photovoltaic power generation, the higher their willingness to pay for photovoltaic power generation. The main reason is that the price rationality reflects that the value and supply and demand are in a balanced state, and a reasonable electricity price is not only conducive to promoting the development of economic production, but also compatible with the affordability of consumers. Therefore, the more reasonable the electricity price, the higher the public’s willingness to pay for photovoltaic power generation [60].

The use safety has a significant positive impact on public photovoltaic power generation WTP_1_, WTP_2_ and WTP_3_, which indicates that the public thinks that the safer the use of photovoltaic power generation, the higher their willingness to pay for photovoltaic power generation. The main reason is that the security and stability of electric power is the fundamental guarantee to ensure the perfection of basic public facilities. Only the security of electric power use can protect public life from being threatened, and then the higher the use safety of photovoltaic power generation, the higher the public willingness to pay [61, 62].

Supply stability has a significant negative impact on public photovoltaic power generation WTP_1_, WTP_2_ and WTP_3_, which indicates that the public thinks that the more stable the supply of photovoltaic power generation, the lower their willingness to pay. The likely reason for this is that PV power supply stabilization both ensures a stable supply of clean energy generation and improves the transparency of PV power supply, facilitating residents’ better understanding of information such as tariff fluctuations and electricity metering methods in the PV power supply chain network, which is perceived by residents as reducing PV power operation costs. Therefore, residents’ willingness to pay decreases with the reduction of PV power generation operating costs [63].

#### 4.1.5 Impact of energy regulation on public willingness to pay for photovoltaic power generation

The recognition degree of energy product tax increase and consumption restriction has a significant positive impact on the public’s willingness to pay for photovoltaic power generation, indicating that the more the public knows about energy product tax increase and consumption restriction, the higher their willingness to pay for photovoltaic power generation will be. The possible reason is that the more the public recognizes energy product tax increase and consumption restriction, the better they can interpret the knowledge of energy product tax increase, and the stronger their willingness to realize low-carbon economic transformation and actively adopt clean energy to protect the environment, so their willingness to pay for photovoltaic power generation is higher [64].

### 4.2 Instrumental variable estimation

Table 6 reports the empirical results of using the exposure degree of nine-year compulsory education as a instrumental variable of respondents’ education level. The measurement results show that the instrumental variables in the first stage have a significant positive impact on the education level at the level of 1%, which meets the basic requirements of instrumental variables. When the public photovoltaic power generation is WTP1, the second stage shows that the P values of Durbin test and Wu-Hausman test are 6.981 and 6.952, respectively. At the level of 1%, the education level is considered as an endogenous variable. The second stage results show that the P value of Wald’s exogenous test is 0.000, and at the level of 1% significance, the original hypothesis that the education level is an exogenous variable is rejected. At the same time, the first-stage model estimates that the F-statistic value is 96.67, which is greater than the critical value of 10, and the second-stage Minimum eigenvalue statistic value is 241.938, which is greater than the critical value of 10% given by Stock-Yogo. Therefore, the instrumental variables selected in this paper are not weak instrumental variables. According to the estimation of the second stage model, the coefficient of public willingness to pay is positive, which is significant at the level of 1% respectively. Therefore, instrumental variables have a good explanatory power in this model. Similarly, instrumental variables can also explain endogenous, exogenous and weak instrumental variables when public photovoltaic power generation is WTP2 and WTP3, so I won’t repeat them here.

**Table 6:** Estimated values in IV.

	WTP_1_		WTP_2_		WTP_3_	
**educational level**	——	4.897***(1.478)	——	5.006***(1.685)	——	5.458***(1.990)
**instrumental variable**	0.768***(0.049)	——	0.768***(0.049)	——	0.768***(0.049)	——
**Control variable**	control	control	control	control	control	control
**constant**	3.242***(0.198)	-15.486**(6.640)	3.242***(0.199)	-16.513**(7.572)	3.242***(0.199)	-19.991**(8.943)
**F statistics**	96.67***		96.67***		96.67***	
**P value of Wald**	0.000***		0.000***		0.000***	
**Minimum eigenvalue statistics**	241.938		241.938		241.938	
**Durbin test p value**	6.981***		4.401**		3.508*	
**Wu-Hausman p value**	6.952***		4.377**		3.488*	
**Sample size**	2363	2363	2363	2363	2363	2363

Note:

* * *, * * and * are significant at the confidence level of 1%, 5% and 10% respectively; Numbers in brackets are standard errors.

The results show that the higher the education level, the greater the WTP of public photovoltaic power generation, and the causal relationship between the higher the education level and the greater the WTP of photovoltaic power generation is determined. The WTP of photovoltaic power generation will increase by 4.897 CNY, 5.006 CNY and 5.458 CNY for each level of education of respondents.

### 4.3 Cluster sample estimation

This part analyzes the causal relationship between education and willingness to pay for solar photovoltaic power generation in different regions of eastern, central and western China. Tables 7–9 show the Heckman two-stage regression of different willingness to pay in eastern, central and western China. The inverse Mills ratio at different levels of willingness to pay in the eastern, central and western regions has passed the significance test, so it is appropriate to use Heckman’s two-stage estimation.

**Table 7:** Results of eastern regression.

	WTP_1_		WTP_2_		WTP_3_	
eastern region	Payment equation	Selection equation	Payment equation	Selection equation	Payment equation	Selection equation
**educational level**	4.066*** (1.303)	0.095** (0.037)	4.466*** (1.492)	0.095** (0.037)	4.581*** (1.564)	0.095** (0.037)
**constant**	18.028 (17.032)	-0.677 (0.448)	18.880 (20.352)	-0.679 (0.448)	18.637 (22.442)	-0.681 (0.448)
**Inverse Millsby**	3.528*** (0.031)		3.634*** (0.031)		3.639*** (0.031)	
**Sample size**	904		904		904	

Note:

* * *, * * and * are significant at the confidence level of 1%, 5% and 10% respectively; Numbers in brackets are standard errors.

**Table 8:** Regression results in the middle.

	WTP_1_		WTP_2_		WTP_3_	
middle region	Payment equation	Selection equation	Payment equation	Selection equation	Payment equation	Selection equation
**educational level**	4.759***(1.340)	0.050(0.042)	4.541***(1.340)	0.050(0.042)	4.288***(1.340)	0.050(0.042)
**constant**	-7.649(21.887)	-2.330***(0.473)	-7.398(23.489)	-2.331***(0.474)	-5.407(23.319)	-2.331***(0.474)
**Inverse Millsby**	3.539***(0.030)		3.534***(0.030)		3.535***(0.030)	
**Sample size**	945		945		945	

Note:

* * *, * * and * are significant at the confidence level of 1%, 5% and 10% respectively; Numbers in brackets are standard errors.

**Table 9:** Results of western regression.

	WTP_1_		WTP_2_		WTP_3_	
West region	Payment equation	Selection equation	Payment equation	Selection equation	Payment equation	Selection equation
**educational level**	2.539**(1.071)	0.096(0.061)	4.629***(1.357)	0.097(0.062)	6.194***(1.737)	0.097(0.062)
**constant**	-4.720(9.929)	-0.630(0.605)	-5.339(12.501)	-0.652(0.607)	-8.778(15.879)	-0.657(0.608)
**Inverse Millsby**	3.070***(0.045)		3.306***(0.044)		3.555***(0.043)	
**No.**	513		513		513	

Note:

* * *, * * and * are significant at the confidence level of 1%, 5% and 10% respectively; Numbers in brackets are standard errors.

Table 7 shows that the second stage regression results show that the education level has a significant positive impact on the willingness to pay for photovoltaic power generation at the level of 1%, indicating that the willingness to pay for photovoltaic power generation in the eastern region has increased significantly with the improvement of public education level. The reason for this phenomenon is that the economy in the eastern region is relatively developed, receiving higher education means high income, and the public in the eastern region has strong economic ability and willingness to pay for photovoltaic power generation to achieve positive externalities of the environment [65].

Table 8 shows that the second stage regression results show that the education level has a significant positive impact on the willingness to pay for photovoltaic power generation at the level of 1%, indicating that the willingness to pay for photovoltaic power generation has increased significantly with the improvement of public education level in the central region. The reason for this phenomenon is that the Chinese government attaches great importance to the development of higher education in the central region. In July, 2010, the Central Committee of the Communist Party of Vietnam and the State Council promulgated the Outline of the National Medium-and Long-Term Education Reform and Development Plan (2010–2020), which proposed to revitalize higher education in central China to solve the problem of backward education in central China. For this reason, the public in central China can enjoy high-quality higher education, and the public in this area has a high level of awareness of clean energy use, which leads to a strong willingness to pay for photovoltaic power generation [66].

Table 9 shows that the results of the second stage regression show that the education level has a significant positive impact on the willingness to pay for photovoltaic power generation, indicating that the willingness to pay for photovoltaic power generation has increased significantly with the improvement of the public education level in the western region. The reason for this phenomenon is that the western region of China has a vast territory, sufficient sunlight and a broad market to develop photovoltaic industry, and the state encourages the development of photovoltaic industry in the western region by tax incentives. Then, the higher the level of public education in the western region, the faster it will get the information of low-cost photovoltaic power generation, and the stronger its willingness to pay for photovoltaic power generation [67].

From Tables 7–9, it can be found that at WTP1, the regional distribution of higher education’s influence effect is in the order of central (4.759) > eastern (4.066) > western (2.539) respectively; At WTP2, the regional distribution order of the influence effect of higher education from big to small is western (4.629) > central (4.541) > eastern (4.466); At WTP3, the regional distribution order of the influence effect of higher education from big to small is western (6.194) > eastern (4.581) > central (4.288).

### 4.4 Instrumental variable estimation of cluster regression

Tables 10–12 report the empirical results of using nine-year compulsory education exposure as a instrumental variable of respondents’ education level in the eastern, central and western regions. Table 10 The measurement results show that the instrumental variables in the first stage have a significant positive impact on the education level at the level of 1%, which meets the basic requirements of instrumental variables. When the public photovoltaic power generation is WTP1, the second stage shows that the P values of Durbin test and Wu-Hausman test are 3.327 and 3.276, respectively, and the education level is considered as an endogenous variable at the level of 10%. The second stage results show that the P value of Wald’s exogenous test is 0.001, and the original hypothesis that education level is an exogenous variable is rejected at the level of 1% significance. At the same time, the first-stage model estimates that the F-statistic value is 29.23, which is greater than the critical value of 10, and the second-stage Minimum eigenvalue statistic value is 100.261, which is greater than the critical value of 16.38 at the 10% level given by Stock-Yogo. Therefore, the instrumental variables selected in this paper are not weak instrumental variables. According to the estimation of the second stage model, the coefficient of public willingness to pay is positive, which is significant at the level of 1% respectively. Therefore, instrumental variables have a good explanatory power in this model. Similarly, the instrumental variables in Tables 11 and 12 can also explain the endogenous, exogenous and weak instrumental variables when the public’s willingness to pay is WTP1, WTP2 and WTP3, so I won’t repeat them here.

**Table 10:** Estimated value of eastern regression IV.

	WTP_1_		WTP_2_		WTP_3_	
eastern region	First-stage	Second-stage	First stage	Second-stage	First stage	Second-stage
**educational level**	——	7.540***(2.703)	——	8.343***(3.042)	——	8.343***(3.042)
**instrumental variable**	0.822***(0.082)	——	0.822***(0.082)	——	0.822***(0.082)	——
**F statistics**	29.23		29.23		29.23	
**The value of Wald**	0.001***		0.001***		0.001***	
**Minimum eigenvalue statistics**	100.261		100.261		100.261	
**Durbin test p value**	3.327*		3.030*		3.030*	
**Wu-Hausman p value**	3.276*		2.983*		2.983*	
**Sample size**	904		904		904	

Note:

* * *, * * and * are significant at the confidence level of 1%, 5% and 10% respectively; Numbers in brackets are standard errors.

**Table 11:** Estimated value of central regression IV.

	WTP1		WTP2		WTP3	
Middle region	First stage	Second-stage	First stage	Second-stage	First stage	Second-stage
**educational level**	——	9.637***(3.415)	——	10.775***(3.479)	——	11.758***(3.567)
**instrumental variable**	0.630***(0.074)	——	0.630***(0.074)	——	0.630***(0.074)	——
**constant**	2.767***(0.337)	-33.484**(13.584)	2.767***(0.337)	-39.301***(13.838)	2.767***(0.337)	-43.619***(14.187)
**F statistics**	33.08		33.08		33.08	
**The value of Wald**	0.000***		0.000***		0.000***	
**Minimum eigenvalue statistics**	71.798		71.798		71.798	
**Durbin test p value**	4.427**		6.104**		7.670*	
**Wu-Hausman test p value**	4.368**		6.033**		7.594*	
**Sample size**	945		945		945	

Note:

* * *, * * and * are significant at the confidence level of 1%, 5% and 10% respectively; Numbers in brackets are standard errors.

**Table 12:** Estimated value of western regression IV.

	WTP1		WTP2		WTP3	
the west	First stage	Second-stage	First stage	Second-stage	First stage	Second-stage
**educational level**	——	12.723**(5.069)	——	15.740***(6.282)	——	17.993**(7.798)
**instrumental variable**	0.309***(0.074)	——	0.309***(0.074)	——	0.309***(0.074)	——
**constant**	3.274***(0.378)	-38.862**(18.256)	3.274***(0.378)	-46.088**(22.625)	3.274***(0.378)	-52.259*(28.082)
**F statistics**	21.64		21.64		21.64	
**The value of Wald**	0.000***		0.000***		0.000***	
**Minimum eigenvalue statistics**	17.499		17.499		17.499	
**Durbin test p value**	5.947**		4.680**		3.399*	
**Wu-Hausman p value**	5.817**		4.567**		3.308*	
**Sample size**	513		513		513	

Note:

* * *, * * and * are significant at the confidence level of 1%, 5% and 10% respectively; Numbers in brackets are standard errors.

The results show that the higher the education level in the eastern region, the greater the WTP of public photovoltaic power generation, and the causal relationship between the higher the education level and the greater the WTP of photovoltaic power generation is determined. The WTP of photovoltaic power generation increased by 7.540 CNY, 8.343 CNY and 8.343 CNY respectively for each level of education of respondents.

The results show that the higher the education level in central China, the greater the WTP of public photovoltaic power generation, and the causal relationship between the higher the education level and the greater the WTP of photovoltaic power generation is determined. The WTP of photovoltaic power generation will increase by 9.637 CNY, 10.775 CNY and 11.758 CNY for each level of education of respondents.

The results show that the higher the education level in the western region, the greater the WTP of public photovoltaic power generation, and the causal relationship between the higher the education level and the greater the WTP of photovoltaic power generation is determined. The WTP of photovoltaic power generation will increase by 12.723 CNY, 15.740 CNY and 17.993 CNY for each level of education of respondents.

### 4.5 Analysis of action mechanism

This part examines the potential moderating factors between education level and willingness to pay. The results are shown in Table 13. For the interaction between education and moderating factors, the positive influence of education level is smaller in the group with higher understanding of ladder price, but larger in the group with male, older than 45, healthier body, higher income and safer electricity consumption.

**Table 13:** Analysis of mechanism of action.

	WTP1	WTP2	WTP3
**Education × Men**	2.227**(1.076)	2.840**(1.235)	3.487**(1.463)
**Education × Age 45+**	2.642*(1.493)	3.034*(1.715)	3.435*(2.031)
**Education × Health**	0.687***(0.163)	0.725***(0.187)	0.790***(0.221)
**Education × Marriage**	-0.387(1.160)	-0.777(1.332)	-0.977(1.578)
**Education x family size**	-0.113(0.427)	-0.155(0.491)	-0.091(0.581)
**Education x income**	0.000***(4.57e-06)	0.000***(5.25e-06)	0.000***(6.21e-06)
**Education × Understanding of Ladder Price**	-1.146**(0.573)	-1.493**(0.658)	-1.772**(0.779)
**Education × Understanding of Peak and Valley Electricity Price**	0.104(0.584)	0.260(0.671)	0.469(0.794)
**Education × Understanding of PV Power Station Subsidy**	0.731(0.696)	0.324(0.799)	0.042(0.946)
**Education× Price Rationality**	-0.105(0.118)	-0.111(0.136)	-0.122(0.161)
**Education × Use Safety**	1.100**(0.529)	1.203**(0.608)	1.290*(0.720)
**Education × Supply Stability**	-0.831(0.529)	-0.769(0.607)	-0.749(0.719)
**Education × Energy Products Tax Increase and Consumption Restriction**	0.772(0.765)	1.177(0.879)	1.411(1.041)
**Education × Compulsory Policy Restricting Energy Consumption**	-0.169(0.790)	-0.174(0.907)	-0.211(1.074)
**Sample size**	2363	2363	2363

Note:

* * *, * * and * are significant at the confidence level of 1%, 5% and 10% respectively; Numbers in brackets are standard errors.

The reason may be that in China, men, the elderly, healthy people and high-income groups have a high social status and are respected in the whole society [68], and the payment cost of photovoltaic power generation accounts for a low marginal cost of family income. In addition, electricity safety means that the environment of family residential quarters is relatively high-grade, and the public is willing to pay clean energy to keep the residential environment clean and tidy. Families with high economic conditions and social status will increase the payment of photovoltaic power generation. The positive influence of education level is smaller in the group with higher understanding of ladder price, but larger in the group with male, older than 45, healthier, higher income and safer electricity use.

Ladder electricity price is the electricity consumption system that mainly takes care of low-income groups to maintain the lowest living standard, and it is also called "poor people’s electricity price". In addition, most of the groups who know better about ladder electricity price are low-income groups, and they may think that photovoltaic power generation will increase electricity bill, so those who know better about ladder electricity price are unwilling to pay photovoltaic power generation.

## 5. Calculation of social and economic value of photovoltaic power generation

Study the key factors of the public’s willingness to pay for photovoltaic power generation is to measure the public’s average willingness to pay, and on this basis, estimate the total social and economic value of photovoltaic power generation. According to formula (1), this paper uses the expectation calculation method in Heckman two-stage estimation to calculate the public’s expected value of the willingness to pay for photovoltaic power generation. When the national public’s willingness to pay for photovoltaic power generation is WTP1, the expected value is 10.63 CNY per household per month, that is, 127.56 CNY per household per year; When the public willingness level of photovoltaic power generation is WTP2, its expected value is 13.15 CNY per household per month, that is, 157.8 CNY per household per year; When the public willingness level of photovoltaic power generation is WTP3, its expected value is 15.76 CNY per household per month, that is, 189.12 CNY per household per year; According to the data of China Demographic and Employment Statistical Yearbook 2018, it is pointed out that there are 375,187 households in China in 2017. According to this, it can be calculated that the estimated total socio-economic value of photovoltaic power generation under different WTPs is 2,838.03 trillion CNY, 3,516.75 trillion CNY and 4,240.44 trillion CNY.

When the willingness level of public photovoltaic power generation in eastern China is WTP1, its expected value is 14.08 CNY per household per month, that is, 168.96 CNY per household per year; when the public willingness level of photovoltaic power generation is WTP2, its expected value is 17.23 CNY per household per month, that is, 206.76 CNY per household per year; When the public willingness level of photovoltaic power generation is WTP3, its expected value is 17.34 CNY per household per month, that is, 208.08 CNY per household per year; According to the 2018 Yearbook of China’s Population and Employment Statistics, there were 147,069 households in eastern China in 2017, which can be calculated. Based on this, it can be calculated that the total social and economic value of photovoltaic power generation in eastern China under different WTPs is estimated to be 1,466.08 trillion CNY, 1,794.07 trillion CNY and 1,805.52 trillion CNY.

When the willingness level of public photovoltaic power generation in central China is WTP1, its expected value is 9.91 CNY per household per month, that is, 118.92 CNY per household per year; When the public willingness level of photovoltaic power generation is WTP2, its expected value is 11.58 CNY per household per month, that is, 138.96 CNY per household per year; When the public willingness level of photovoltaic power generation is WTP3, its expected value is 13.23 CNY per household per month, that is, 158.76 CNY per household per year; According to the relevant data of China’s Statistical Yearbook of Population and Employment in 2018, there were 127,845 households in central China in 2017. Based on this, it can be calculated that the estimated total social and economic value of photovoltaic power generation in central China under different WTPs is 897.00 trillion CNY, 1,048.16 trillion CNY and 1,197.50 trillion CNY.

When the willingness level of public photovoltaic power generation in western China is WTP1, its expected value is 7.98 CNY per household per month, that is, 95.77 CNY per household per year; When the public willingness level of photovoltaic power generation is WTP2, its expected value is 10.20 CNY per household per month, that is, 122.4 CNY per household per year; When the public willingness level of photovoltaic power generation is WTP3, its expected value is 12.7 CNY per household per month, that is, 152.4 CNY per household per year; According to the relevant data of China Demographic and Employment Statistical Yearbook 2018, there were 95,668 households in western China in 2017. According to this, it can be calculated that the estimated total social and economic value of photovoltaic power generation in western China under different WTPs is 557.06 trillion CNY, 653.79 trillion CNY and 903.95 trillion CNY.

## 6. Conclusion

In this paper, we utilize data from the 2018 China Integrated Social Survey (CISS) to validate the endogenous issues between education and clean power using the implementation of the compulsory education law, and to determine the causal relationship between education level and clean power with different WTPs in eastern, central, and western China, and to further validate the mechanism of the role of education level and clean power WTP. The results found that both the Heckman model and the IV tool identified a positive effect of education level on WTP clean electricity. For every increase in the education level of respondents in the eastern region, the WTP of PV power increased by $7.540, $8.343 and $8.343, respectively. For each level of increase in the education level of the respondents in the central region, the PV WTP increases by $9.637, $10.775, and $11.758, respectively. For each level increase in the education level of respondents in the western region, the PV WTP increases by $12.723, $15.740 and $17.993, respectively. The mechanism of action found the positive impact of education level to be smaller in the group with higher level of understanding of step tariffs and larger in the group of males, older than 45 years old, healthier, higher income and safer electricity usage. The total socio-economic value of PV power generation in the country ranges from $2,838.03 trillion to $4,240.44 trillion. The total socio-economic value of PV power generation in the East is 1,466.08 trillion yen ~ 1,805.52 trillion yen. The total socio-economic value of photovoltaic power generation in the central part of the country is 897.00 trillion yen ~ 1197.50 trillion yen. The total socio-economic value of photovoltaic power generation in the west is 557.06 trillion yuan to 903.95 trillion yuan.

In response to these findings, we offer the following insights for policymakers: this paper finds that higher education significantly increases clean power WTP, suggesting that strengthening higher education should be considered an important tool for promoting clean power adoption in developed countries. The study in this paper supports previous research findings that education increases clean electricity adoption behavior [2]. Family characteristics play an important role in clean electricity adoption. Therefore, this paper is crucial to increase clean electricity adoption through education. The successful transition from administrative to market-based environmental instruments depends not only on top-down government policies, but also on a steady increase in the public’s environmental awareness and willingness to contribute at the individual level [7]. Financial expenditure on education contributes to environmental protection in WTP and the spillover effect is significant, and government expenditure on environmental protection may squeeze each other with financial expenditure on education, then the government should pay attention to the public’s environmental awareness and ability of family characteristics and promote environmental protection concepts to all aspects of life to reduce the burden of financial payments.

This paper finds that the average WTP in the east is higher than that in the national, central and western regions. In addition, the IV estimation results indicate that the impact of education on clean power WTP in the central and western regions is much higher than that in the eastern region, which suggests that there is a great potential for the improvement of clean power WTP in the central and western regions. However, there is a serious imbalance in the distribution of education resources among the eastern, central and western chiropractic regions of China, with the investment in education resources in the eastern region significantly higher than that in the central and western regions [69]. According to the environmental Kuznets curve synthesis [70], developed regions will gradually phase out production capacity in polluting industries, and less developed regions will take over production capacity to promote local economic growth. This transfer of technology and pollution is happening in China today [71]. The central and western regions of China will face more challenges in pollution control and environmental protection, and improving the education level in the central and western regions will reduce environmental pollution. The government can raise funds to compensate for the higher cost of cleaner electricity generation through "price discrimination" by adding different surcharges to electricity tariffs in different regions. Energy foundations, green power funds are feasible ways to raise funds to support renewable energy generation. Therefore, comprehensive policies and measures should be put in place to collect and utilize the funds to ensure that they are actually used to finance renewable energy generation.

## Supporting information

S1 File (XLS)
